# Characteristics of Group A *Streptococcus* Strains Circulating during Scarlet Fever Epidemic, Beijing, China, 2011

**DOI:** 10.3201/eid1906.121020

**Published:** 2013-06

**Authors:** Peng Yang, Xiaomin Peng, Daitao Zhang, Shuangsheng Wu, Yimeng Liu, Shujuan Cui, Guilan Lu, Wei Duan, Weixian Shi, Shuang Liu, Jing Li, Quanyi Wang

**Affiliations:** Beijing Center for Disease Prevention and Control, Beijing, China; and Capital Medical University School of Public Health, Beijing

**Keywords:** scarlet fever, pharyngitis, group A Streptococcus, GAS, emm type, superantigen, antimicrobial drug susceptibility, bacteria, epidemic, surveillance, Beijing, People’s Republic of China, China

## Abstract

Scarlet fever is one of a variety of diseases caused by group A *Streptococcus* (GAS). During 2011, a scarlet fever epidemic characterized by peak monthly incidence rates 2.9–6.7 times higher than those in 2006–2010 occurred in Beijing, China. During the epidemic, hospital-based enhanced surveillance for scarlet fever and pharyngitis was conducted to determine characteristics of circulating GAS strains. The surveillance identified 3,359 clinical cases of scarlet fever or pharyngitis. GAS was isolated from 647 of the patients; 76.4% of the strains were type *emm*12, and 17.1% were *emm*1. Almost all isolates harbored superantigens *speC* and *ssa*. All isolates were susceptible to penicillin, and resistance rates were 96.1% to erythromycin, 93.7% to tetracycline, and 79.4% to clindamycin. Because *emm*12 type GAS is not the predominant type in other countries, wider surveillance for the possible spread of *emm*12 type GAS from China to other countries is warranted.

*Streptococcus pyogenes*, also known as group A *Streptococcus* (GAS), is a common human pathogen that can induce a wide spectrum of diseases, ranging from noninvasive diseases, such as pharyngitis, scarlet fever, and impetigo, to invasive diseases, such as erysipelas, cellulitis, pneumonia, bacteremia, necrotizing fasciitis, and toxic shock syndrome. Moreover, GAS can cause rheumatic fever and acute poststreptococcal glomerulonephritis (*1*,*2*). In the late1980s, a change in the epidemiology of invasive GAS diseases and the emergence of streptococcal toxic shock syndrome were documented (*3*,*4*), and the current number of invasive GAS disease cases worldwide is high (*2*).

Many virulence factors contribute to the pathogenesis of GAS diseases (*1*,*5*). However, the matrix (M) protein, encoded by the *emm* gene, has the most critical role, mainly by antiphagocytic mechanisms (*6*,*7*), and the amino-terminal region of M protein is the most promising target for designing a vaccine (*8*,*9*). *emm* gene sequencing is a standard method for typing the M protein (*10*), but the distribution of *emm* types varies greatly by geographic location, time, and collection site of clinical specimens (*9*,*11*–*14*).

Streptococcal pyrogenic exotoxins also play a major role in the pathogenesis of GAS infections by acting as superantigens. When these exotoxins cross-link major histocompatibility complex class II molecules and T cell receptors, they trigger intense activation of a subset of T cells within a specific β-chain variable region. This process induces a tremendous release of a series of cytokines and may lead to cell, tissue, and organ damage (*15*,*16*).

Several antimicrobial drugs effectively treat GAS infections (*1*). In recent years, however, considerable attention has been given worldwide to the issue of antimicrobial drug–resistant GAS. Macrolide-resistant GAS strains have been isolated from various regions of the world (*17*–*19*). Macrolides are used as an alternative treatment for GAS in patients allergic to penicillin, and clindamycin, in combination with β-lactam antimicrobial drugs, is a recommended treatment for invasive GAS disease (*20*). Thus, it is critical that surveillance for macrolide- and clindamycin-resistant GAS be continued.

In China, scarlet fever is the only GAS disease reported by the National Notifiable Infectious Disease Surveillance System (NNIDSS) (*21*). According to NNIDSS, the incidence of scarlet fever in Beijing, China, before 2011 had persistently remained within normal threshold limits. However, in late spring 2011, an epidemic of scarlet fever occurred in Beijing and many other regions of China. In response to the epidemic, enhanced surveillance for GAS diseases was conducted in Beijing during May–July 2011 to determine characteristics of the circulating GAS strains.

## Methods

### National Notifiable Infectious Disease Surveillance System

China established NNIDSS in 2004, after the 2003 outbreak of severe acute respiratory syndrome. At that time, NNIDSS covered 37 infectious diseases, which were classified into 3 categories (A–C), in a descending order according to disease severity; scarlet fever belonged to category B.

A clinical case of scarlet fever is defined as acute illness onset with fever, pharyngitis, and sandpaper-like red rash with or without strawberry tongue, Pastia lines, or circumoral pallor. In China, clinicians and hospitals are to report clinical cases of scarlet fever to NNIDSS within 6 hours of diagnosis. To respond to the 2011 epidemic of scarlet fever and to monitor its severity, each clinical case of scarlet fever reported in Beijing was followed for 3 weeks after the onset of disease.

### Enhanced Surveillance for GAS Diseases

Enhanced surveillance for GAS diseases was conducted in the pediatric clinics of 36 hospitals within Beijing’s 18 districts during May–July 2011. The surveillance system was designed and managed by the Beijing Center for Disease Prevention and Control (Beijing CDC). The Beijing CDC laboratory and 18 collaborating district laboratories were involved. The study was approved by the Institutional Review Board and the Human Research Ethics Committee of Beijing CDC.

The surveillance included children with scarlet fever or pharyngitis diagnosed by clinicians in the surveillance hospitals. All children with scarlet fever were invited to participate in the study after their parent(s)/guardian(s) gave informed consent. Each week, 10 children with pharyngitis were randomly selected from each hospital to participate in the study. Trained clinic staff used a standardized questionnaire to collect information (e.g., sex, age, date of illness onset, date medical care was sought, clinical symptoms and signs, and antimicrobial drug treatment) for each study participant. In addition, at each clinic, trained personnel obtained pharyngeal swab samples from study participants, and, the same day, a designated hospital staff member collected and sent all specimens to the corresponding district laboratory. The collaborating district laboratories isolated and identified GAS strains and then sent the isolates to Beijing CDC for *emm* typing, superantigen determination, and antimicrobial drug susceptibility testing. Patients with scarlet fever or pharyngitis from whom GAS was isolated were identified as confirmed GAS patients.

### Isolation and Identification of GAS Strains

After pharyngeal swab samples arrived at a collaborating district laboratory, they were immediately spread onto 5% sheep blood agar plates and incubated overnight at 37°C in 5% CO_2_. We tested β-hemolytic isolates for susceptibility to bacitracin and used the Streptococcal Grouping Kit (Oxoid Ltd., Basingstoke, UK) to determine the Lancefield group for each isolate.

### *emm* Typing

All GAS isolates were subjected to *emm* typing, as described (*22*). We extracted DNA and amplified and sequenced the 5′ region of the *emm* gene by using recommended primers and cycling conditions (*22*). We aligned the sequence of the 5′ region of the *emm* gene with sequences in the Blast-*emm* database (www.cdc.gov/ncidod/biotech/strep/strepblast.htm; Centers for Disease Control and Prevention, Atlanta, GA, USA). The *emm* type and subtype of GAS isolates were identified on the basis of the 90 bases encoding the N terminal 30 residues of the processed M protein and the exact 150 base sequences encoding the N terminal 50 residues of the mature M protein, respectively. The fairly conserved 30 bases encoding the last 10 residues of the M protein signal sequence were referred to for identifying the start of the sequence encoding the mature M protein.

### Superantigen Detection 

Thirteen superantigens (*speA*–*speC*, *speF*–*speM*, *smeZ*, and *ssa*) were detected by subjecting each GAS isolate to real-time PCR. Specific PCR primers were used to amplify the gene of each superantigen in a 40-μL real-time PCR reaction system under the following cycling condition: 2 min at 94°C, followed by 40 cycles of 15 s at 93°C and 60 s at 55°C. Superantigen profiles were investigated for various *emm* types of GAS isolates.

### Antimicrobial Drug Susceptibility Testing

We used the VITEK2 Compact (bioMérieux, Marcy l'Etoile, France) to test all GAS isolates for susceptibility to penicillin, ampicillin, quinupristin-dalfopristin, linezolid, vancomycin, tigecycline, levofloxacin, erythromycin, clindamycin, and tetracycline. We estimated the minimal inhibitory concentration of each antimicrobial drug for individual GAS isolates and determined the corresponding susceptibility according to the 2011 criteria of the Clinical and Laboratory Standards Institute (*23*).

### Statistical Analysis

We entered data by using Microsoft Excel 2003 software (Microsoft Corp., Redmond, WA, USA) and analyzed data by using the SPSS 16.0 statistical package (SPSS Inc., Chicago, IL, USA). The mean and SD were calculated for continuous variables, and percentages were calculated for categorical variables. Differences in distributions of *emm* types and superantigens of GAS isolates were compared between subgroups of participants by using the χ^2^ test or Fisher exact test. In addition, we compared antimicrobial drug susceptibilities by strain *emm* type by using the χ^2^ test. We used multivariate unconditional logistic regression analyses to determine factors associated with various clinical signs of scarlet fever in patients with confirmed GAS infection. All statistical tests were 2-sided, and statistical significance was defined as p<0.05.

## Results

### Comparison of the 2011 Epidemic and 2006–2010 Cluster Outbreaks

According to NNIDSS, the annual number of scarlet fever cases in Beijing during 2006–2010 ranged from 1,193 to 2,264, and annual incidence rates ranged from 7.0 cases to 14.3 cases/100,000 population. In 2011, however, the number of scarlet fever cases in Beijing rose to 6,152, and the incidence rate rose to 31.4 cases/100,000 population. Peak monthly incidence rates in 2011 were 2.9–6.7 times those in 2006–2010. During the epidemic and during 2006–2010, scarlet fever cases peaked twice yearly: 1 peak occurred in early summer, and a second, less pronounced peak occurred in winter, except in 2010, when the winter peak was more pronounced (Figure, panel A). 

**Figure:**
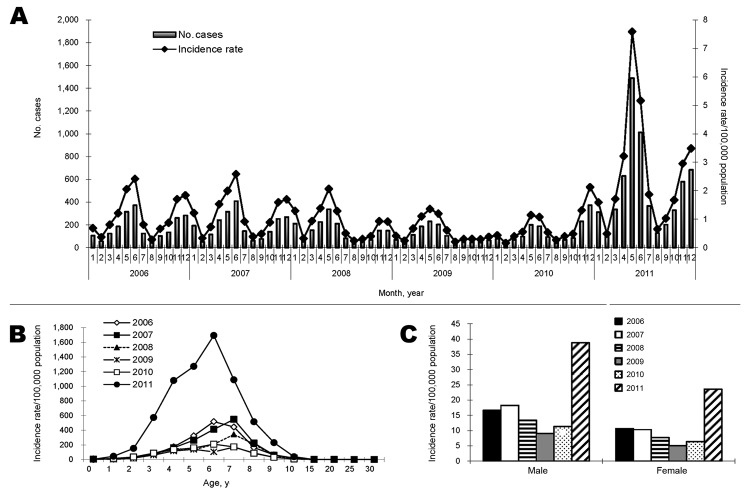
Scarlet fever incidence, Beijing, China, 2006–2011, as reported in the National Notifiable Infectious Disease Surveillance System. A) Number of cases and incidence rate by month. B) Incidence rate by age. C) Incidence rate by sex.

Scarlet fever primarily affected children 3–8 years of age during the epidemic and nonepidemic years (Figure, panel B). During these years, the ratio of males to females in Beijing was 1.5:1.8, respectively, and the annual incidence of scarlet fever was persistently higher among males than females (Figure, panel C).

In Beijing, a cluster of scarlet fever was defined as onset of >2 clinical cases within a 7-day period in a school or kindergarten. According to this definition, 37–131 clusters (85–316 cases) occurred during 2006–2010 in Beijing, and 401 clusters (1,116 cases) occurred during 2011. In addition, case-patient follow-up showed that all cases reported during May–December, 2011, were fully resolved 3 weeks after illness onset without complications.

### Characteristics of Children Enrolled in Enhanced Surveillance

A total of 3,359 children, representing 972 clinical cases of scarlet fever and 2,387 cases of pharyngitis, were enrolled in enhanced surveillance for GAS disease. Of the 3,359 enrollees, ≈41.0% were female. The mean age was 5.8 years (SD ± 3.3 years). The mean interval from illness onset to first medical visit was 1.5 days (SD ± 1.5 days). Approximately 41.2% of the children received antimicrobial drugs at home before seeking medical care.

Of the 3,359 enrollees, 647 (19.2%) were confirmed to have GAS infection. GAS was isolated from clinical samples for 44.2% (430/972) of the children with scarlet fever and from 9.1% (217/2,387) of the children with pharyngitis. 

### *emm* Subtypes and Superantigens

*emm*12 type accounted for 76.4% (494/647) of all GAS isolates. The leading *emm* subtypes were *emm*12.0 (65.7%), *emm*1.0 (16.8%), *emm*12.19 (5.7%), and *emm*12.1 (2.5%). There was a statistically significant difference in the proportion of *emm*12 type GAS in children <5 years of age and those >5 years of age (p = 0.004) and in the proportion of *emm*1 scarlet fever cases and pharyngitis cases (p = 0.042) (Table 1).

**Table 1:** SAgs and *emm* types of group A *Streptococcus* circulating during a scarlet fever epidemic, Beijing, China, 2011*

*emm* type or SAg	No. (%) patients, by age		p value	No. (%) patients, by sex		p value	No. (%) patients, by clinical diagnosis		p value	Total, n = 647
	<5 y, n = 246	>5 y, n = 401		Male, n = 402	Female, n = 245		Scarlet fever, n = 430	Pharyngitis, n = 217		
*emm* type										
12	203 (82.5)	291 (72.6)	**0.004**	303 (75.4)	191 (78.0)	0.453	331 (77.0)	163 (75.1)	0.599	494 (76.4)
1	34 (13.8)	77 (19.2)	0.078	73 (18.2)	38 (15.5)	0.386	83 (19.3)	28 (12.9)	**0.042**	111 (17.1)
Other†	9 (3.7)	33 (8.2)	**0.022**	26 (6.5)	16 (6.5)	0.975	16 (3.7)	26 (12.0)	**<0.001**	42 (6.5)
SAg										
* SpeA*	47 (19.1)	96 (23.9)	0.150	91 (22.6)	52 (21.2)	0.675	99 (23.0)	44 (20.3)	0.427	143 (22.1)
* SpeB*	246 (100.0)	401 (100.0)	NA	402 (100.0)	245 (100.0)	NA	430 (100.0)	217 (100.0)	NA	647 (100.0)
*SpeC*‡	243 (98.8)	400 (99.8)	0.156	398 (99.0)	245 (100.0)	0.303	427 (99.3)	216 (99.5)	1.000	643 (99.4)
*SpeF*‡	245 (99.6)	400 (99.8)	1.000	401 (99.8)	244 (99.6)	1.000	429 (99.8)	216 (99.5)	1.000	645 (99.7)
*SpeG*‡	246 (100.0)	399 (99.5)	0.528	400 (99.5)	245 (100.0)	0.529	428 (99.5)	217 (100.0)	0.554	645 (99.7)
* SpeH*	192 (78.0)	301 (75.1)	0.387	299 (74.4)	194 (79.2)	0.164	326 (75.8)	167 (77.0)	0.747	493 (76.2)
* SpeI*	190 (77.2)	302 (75.3)	0.578	301 (74.9)	191 (78.0)	0.373	327 (76.0)	165 (76.0)	1.000	492 (76.0)
* SpeJ*	47 (19.1)	92 (22.9)	0.249	87 (21.6)	52 (21.2)	0.900	99 (23.0)	40 (18.4)	0.180	139 (21.5)
*SpeK* ‡	1 (0.4)	3 (0.7)	1.000	1 (0.2)	3 (1.2)	0.155	0	4 (1.8)	**0.012**	4 (0.6)
*SpeL* ‡	2 (0.8)	5 (1.2)	0.715	4 (1.0)	3 (1.2)	1.000	2 (0.5)	5 (2.3)	**0.046**	7 (1.1)
* SpeM*	5 (2.0)	9 (2.2)	0.857	8 (2.0)	6 (2.4)	0.697	9 (2.1)	5 (2.3)	0.862	14 (2.2)
*SmeZ*‡	245 (99.6)	400 (99.8)	1.000	401 (99.8)	244 (99.6)	1.000	428 (99.5)	217 (100.0)	0.554	645 (99.7)
* Ssa*	241 (98.0)	391 (97.5)	0.705	394 (98.0)	238 (97.1)	0.477	427 (99.3)	205 (94.5)	**<0.001**	632 (97.7)

*Boldface indicates statistical significance. SAg, superantigen. †Other *emm* types included types 4, 11, 22, 75, and 89. ‡Fisher exact test was used.

All of the GAS isolates harbored *speB*, and almost all possessed *speC*, *speF*, *speG*, *smeZ*, and *ssa*. Approximately 20% of the isolates harbored *speA* or *speJ*, and ≈75% possessed *speH* or *speI*; however, the percentage of isolates that harbored *speK*, *speL*, or *speM* was extremely low (Table 1). A total of 25 profiles of superantigens were found in the GAS isolates. Of the 494 *emm*12 isolates, 411 (83.2%) had the following superantigen profile: *speA* (−), *speB* (+), *speC* (+), *speF* (+), *speG* (+), *speH* (+), *speI* (+), *speJ* (−), *speK* (−), *speL* (−), *speM* (−), *smeZ* (+), *ssa* (+). Of the 111 *emm*1 isolates, 93 (83.8%) had the following superantigen profile: *speA* (+), *speB* (+), *speC* (+), *speF* (+), *speG* (+), *speH* (−), *speI* (−), *speJ* (+), *speK* (−), *speL* (−), *speM* (−), *smeZ* (+), *ssa* (+).

### Antimicrobial Drug Susceptibility

All GAS strains isolated during the scarlet fever epidemic were susceptible to penicillin, ampicillin, quinupristin-dalfopristin, linezolid, vancomycin, and tigecycline, and 96.6% of them were susceptible to levofloxacin. Resistance to erythromycin, tetracycline, and clindamycin was found in 96.1%, 93.7%, and 79.4% of the isolates, respectively. A statistically significant difference was found between the percentage of *emm*1 and *emm*12 strains resistant to clindamycin (87.4% vs. 77.9%, respectively; p = 0.025) but not between the percentage of those resistant to erythromycin (99.1% vs. 96.4%, respectively; p = 0.134) or tetracycline (97.3% vs. 93.1%, respectively; p = 0.097). 

### Factors Associated with Clinical Signs of Scarlet Fever

The odds of having strawberry tongue was higher for GAS-infected study participants <5 years of age (odds ratio [OR] 2.04, 95% CI 1.46–2.83; p < 0.001). The odds of having a red rash was higher for participants infected with an *emm*1 versus *emm*12 type strain (OR 1.63, 95% CI 1.01–2.62; p = 0.046) and for participants <5 years of age (OR 2.52, 95% CI 1.74–3.65; p<0.001). Compared with patients with *emm*12 type strains, those with *emm*1 type strains had a higher probability of having Pastia lines (OR 1.80, 95% CI 1.02–3.16; p = 0.043) or circumoral pallor (OR 2.15, 95% CI 1.17–3.93; p = 0.013) (see Table 2, Appendix, wwwnc.cdc.gov/EID/article/19/6/12-1020-T1.htm, for details).

**Table 2:** Results of multivariate analyses for factors associated with 4 clinical signs of scarlet fever in patients with confirmed GAS infection, Beijing, China, 2011*

Factor	Clinical sign of scarlet fever†														
	Strawberry tongue				Red rash				Pastia lines				Circumoral pallor		
	Yes, n = 293	No, n = 354	OR (95% CI), p value		Yes, n = 430	No, n = 217	OR (95% CI), p value		Yes, n = 79	No, n = 568	OR (95% CI), p value		Yes, n = 63	No, n = 584	OR (95% CI), p value
*emm* type															
12	232 (79.2)	262 (74.0)	Ref		331 (77.0)	163 (75.1)	Ref		57 (72.2)	437 (76.9)	Ref		43 (68.3)	451 (77.2)	Ref
1	51 (17.4)	60 (16.9)	1.03 (0.68–1.58), 0.884		83 (19.3)	28 (12.9)	**1.63 (1.01–2.62), 0.046**		20 (25.3)	91 (16.0)	**1.80 (1.02–3.16), 0.043**		18 (28.6)	93 (15.9)	**2.15 (1.17–3.93), 0.013**
Other‡	10 (3.4)	32 (9.0)	**0.40 (0.19–0.84), 0.016**		16 (3.7)	26 (12.0)	**0.35 (0.18–0.68), 0.002**		2 (2.5)	40 (7.0)	0.40 (0.09–1.72), 0.218		2 (3.2)	40 (6.8)	0.54 (0.12–2.31), 0.402
Age <5 y	138 (47.1)	108 (30.5)	**2.04 (1.46–2.83), <0.001**		193 (44.9)	53 (24.4)	**2.52 (1.74–3.65), <0.001**		35 (44.3)	211 (37.1)	1.40 (0.86–2.27), 0.182		27 (42.9)	219 (37.5)	1.29 (0.75–2.22), 0.356
Female sex	105 (35.8)	140 (39.5)	1.09 (0.78–1.51), 0.630		157 (36.5)	88 (40.6)	1.09 (0.77–1.55), 0.615		36 (45.6)	209 (36.8)	0.65 (0.40–1.05), 0.079		28 (44.4)	217 (37.2)	0.69 (0.40–1.18), 0.171
Interval >2 d§	51 (17.4)	39 (11.0)	**1.87 (1.18–2.97), 0.008**		61 (14.2)	29 (13.4)	1.19 (0.73–1.95), 0.489		7 (8.9)	83 (14.6)	0.58 (0.26–1.31), 0.190		3 (4.8)	87 (14.9)	**0.29 (0.09–0.94), 0.040**

*Four multivariate unconditional logistic regression analyses were separately conducted to examine factors for each clinical sign of scarlet fever. Scarlet fever and pharyngitis patients from whom GAS was isolated were identified as confirmed GAS patients. **Boldface** font indicates statistical significance. GAS, group A *Streptococcus*; OR, odds ratio; Ref, reference. †Data are number (%) patients unless otherwise indicated. ‡Other *emm* types included types 4, 11, 22, 75, and 89. §Interval between illness onset and hospital visit.

## Discussion

We found that an epidemic of scarlet fever occurred in Beijing in 2011. *emm*12 was the predominant type among the circulating GAS strains, and resistance to erythromycin, tetracycline, and clindamycin was high among the isolates.

Although the incidence of scarlet fever in Beijing in 2011 was much higher than that in preceding years, the basic characteristics, including the seasonality of epidemic peaks, the most vulnerable age group, and the difference in susceptibility to infection by sex, did not change. In Beijing, the incidence of scarlet fever usually peaks twice a year in 2 distinct seasons—summer and winter—with the highest peak in early summer; however, it is well-recognized that scarlet fever most commonly occurs in winter/spring in other locations (*24*,*25*). This finding indicates that in China, GAS is transmitted more easily in early summer than in winter, possibly because in early summer, compared with winter, each year an *emm* type that is relatively new to young children sweeps through a population of children that does not have an effective level of population immunity. In addition, the winter peak in 2010 was higher than the summer peak, which implies that this epidemic of scarlet fever started at the end of 2010.

Among the GAS strains we identified circulating in Beijing during 2011, types *emm*12 and *emm*1 were most predominant (≈76% and ≈17%, respectively); these percentages were higher (≈40%) and lower (>40%), respectively, than those reported in China during previous years (*13*,*26*,*27*). Scarlet fever outbreaks also occurred in Hong Kong and Shanghai, China, during 2011, and *emm*12 was the predominant circulating type (*28*–*30*). These findings suggest that some intrinsic factors might have facilitated the spread of *emm*12 strains and led to the epidemic. Two previously unreported genomic insertions (64.9 kb and 46.4 kb) were identified in *emm*12 GAS strains isolated during the 2011 scarlet fever outbreak in Hong Kong. However, analysis of *emm*12 strains isolated during 2005–2010 showed that the insertions were also present in those strains (*28*), and the study concluded that mobile genetic elements, environmental factors, and host immune status might have contributed to the 2011 scarlet fever outbreak in Hong Kong.

*emm*12 is not known to be the exclusively predominant GAS type in other countries (*9*,*31*,*32*), but it is possible that the *emm*12 type strains circulating in China in 2011 could spread to other regions. Therefore, surveillance for the increased presence of *emm*12 strains outside of China is warranted. A 30-valent GAS vaccine that contains the most prevalent *emm* types found in our study (*emm*12 and *emm*1) is under development (*8*); such a vaccine would help prevent and control the spread of GAS diseases in China.

Superantigens in the GAS isolates in our study were similar to those found in isolates from other studies, except for *speC* and *ssa*. In contrast to findings in other studies, we found that almost all *emm*1 GAS strains from the 2011 Beijing epidemic harbored *speC*, and *ssa* became the primary superantigen of *emm*1 and *emm*12 isolates (*11*,*32*,*33*). Consistent with findings in earlier studies in other locations, we found that an extremely low number of GAS isolates harbored *speK*, *speL,* or *speM* (*11*,*33*,*34*).

All GAS isolates from the 2011 Beijing epidemic were susceptible to penicillin, a standard antimicrobial drug for the treatment of scarlet fever. However, >96% and ≈94% of the isolates were resistant to erythromycin and tetracycline, respectively, and ≈80% were resistant to clindamycin. These findings compare with earlier findings of 99.5%, 97.1%, and 99.5% resistance to erythromycin, tetracycline, and clindamycin, respectively (*27*). The extent of the resistance of GAS strains to erythromycin and tetracycline in many other countries has been very low (*11*,*17*,*18*). The high resistance rate in China might be attributed to the overuse of antimicrobial drugs in humans or animals.

In our study, the odds of having strawberry tongue or red rash with GAS infection was higher for younger patients. This could indicate that older patients had acquired partial immunity to GAS from a previous exposure or infection, resulting in milder clinical manifestation of the disease during subsequent infection.

This study had limitations. First, to track case outcomes, we followed up on the scarlet fever case-patients for 3 weeks after illness onset, which may not have allowed sufficient time to capture later-occurring complications; thus, outcome profiles may have been incomplete. Second, the enhanced surveillance in Beijing did not include invasive GAS diseases, so we could not report on all GAS strains circulating during the 2011 scarlet fever epidemic.

The 2011 scarlet fever epidemic in Beijing was characteristic of other scarlet fever epidemics and occurred after an abnormal peak winter incidence of the disease in 2010. *emm*12 type GAS became predominant. The level of GAS resistance to clindamycin was lower than that to erythromycin and tetracycline.
